# Walking performance is positively correlated to calf muscle fiber size in peripheral artery disease subjects, but fibers show aberrant mitophagy: an observational study

**DOI:** 10.1186/s12967-016-1030-6

**Published:** 2016-09-29

**Authors:** Sarah H. White, Mary M. McDermott, Robert L. Sufit, Kate Kosmac, Alex W. Bugg, Marta Gonzalez-Freire, Luigi Ferrucci, Lu Tian, Lihui Zhao, Ying Gao, Melina R. Kibbe, Michael H. Criqui, Christiaan Leeuwenburgh, Charlotte A. Peterson

**Affiliations:** 1 College of Health Sciences and Center for Muscle Biology, University of Kentucky, 900 S Limestone CTW105, Lexington, KY 40536 USA; 2 Division of General Internal Medicine, Department of Medicine, Northwestern University Feinberg School of Medicine, 750 North Lake Shore Drive, 10th Floor, Chicago, 60611 USA; 3 Department of Preventive Medicine, Northwestern University Feinberg School of Medicine, Chicago, IL USA; 4 Department of Neurology, Northwestern University Feinberg School of Medicine, Chicago, IL USA; 5 National Institute on Aging, Baltimore, MD USA; 6 Department of Health Research & Policy, Stanford University, Stanford, CA USA; 7 Department of Surgery, Northwestern University Feinberg School of Medicine, Chicago, IL USA; 8 Jesse Brown Veterans Affairs Medical Center, Chicago, IL USA; 9 Department of Family Medicine and Public Health, University of California at San Diego, La Jolla, CA USA; 10 Department of Aging and Geriatric Research, University of Florida Institute on Aging, Gainesville, FL USA

**Keywords:** Mitochondria, Mitophagy, Fiber type, Calf muscle, Peripheral artery disease

## Abstract

**Background:**

Patients with lower extremity peripheral artery disease (PAD) have decreased mobility, which is not fully explained by impaired blood supply to the lower limb. Additionally, reports are conflicted regarding fiber type distribution patterns in PAD, but agree that skeletal muscle mitochondrial respiration is impaired.

**Methods:**

To test the hypothesis that reduced muscle fiber oxidative activity and type I distribution are negatively associated with walking performance in PAD, calf muscle biopsies from non-PAD (n = 7) and PAD participants (n = 26) were analyzed immunohistochemically for fiber type and size, oxidative activity, markers of autophagy, and capillary density. Data were analyzed using analysis of covariance.

**Results:**

There was a wide range in fiber type distribution among subjects with PAD (9–81 % type I fibers) that did not correlate with walking performance. However, mean type I fiber size correlated with 4-min normal- and fastest-paced walk velocity (r = 0.4940, P = 0.010 and r = 0.4944, P = 0.010, respectively). Although intensity of succinate dehydrogenase activity staining was consistent with fiber type, up to 17 % of oxidative fibers were devoid of mitochondria in their cores, and the core showed accumulation of the autophagic marker, LC3, which did not completely co-localize with LAMP2, a lysosome marker.

**Conclusions:**

Calf muscle type I fiber size positively correlates with walking performance in PAD. Accumulation of LC3 and a lack of co-localization of LC3 with LAMP2 in the area depleted of mitochondria in PAD fibers suggests impaired clearance of damaged mitochondria, which may contribute to reduced muscle oxidative capacity. Further study is needed to determine whether defective mitophagy is associated with decline in function over time, and whether interventions aimed at preserving mitochondrial function and improving autophagy can improve walking performance in PAD.

**Electronic supplementary material:**

The online version of this article (doi:10.1186/s12967-016-1030-6) contains supplementary material, which is available to authorized users.

## Background

Patients with lower extremity peripheral artery disease (PAD), caused by atherosclerosis in the lower extremities, have faster functional decline and higher rates of mobility loss compared to people without PAD [1–7]. As therapeutic advances have not kept pace with the growing burden of disability from this chronic disease [8], new approaches are urgently needed to prevent disability in patients with PAD.

Patients with PAD experience ischemia of lower limb skeletal muscle during walking activity, when metabolic demands exceed oxygen supply, followed by reperfusion during rest. Ischemia has been demonstrated to damage skeletal muscle fibers and impair mitochondrial activity, and reperfusion exacerbates this damage [9–13]. As a result, small studies of PAD participants show quantitative mitochondrial dysfunction in gastrocnemius muscle, with reduced ATP production [11, 14]. Low mitochondrial activity, measured either by respirometry from muscle biopsy specimens or using magnetic resonance spectroscopy, is associated with poorer treadmill walking in people with PAD [9, 14]. Moreover, while mitochondria-specific autophagy, known as mitophagy, has not been directly explored in PAD, electron microscopy of gastrocnemius biopsies in patients with PAD shows pathologic changes in mitochondria [15]. Further, research has shown that gastrocnemius mitochondrial content may predict mortality rate in PAD [16]. While bypass surgery improves walking performance in PAD patients [17], it is associated with a higher degree of morbidity and mortality, and patients may later require additional procedures with a greater risk of limb loss [18]. Therefore, alternative strategies, perhaps targeting mitochondrial health, may be advantageous targets of future therapies for PAD patients.

Human skeletal muscle is characterized by three main fiber types, which vary in fatigue resistance and oxidative potential. Type I, slow-twitch fibers have the greatest mitochondrial density and, therefore, greatest oxidative metabolism, resulting in high resistance to fatigue [19]. Types IIa and IIx are fast-twitch fibers, with IIa having intermediate oxidative activity and fatigability and IIx relying primarily on glycolytic, anaerobic metabolism, fatiguing the most quickly [19]. Muscle fiber type composition, which is normally closely associated with mitochondrial oxidative capacity, is altered in muscle from PAD patients, but reports are contradictory. In patients with PAD, a decrease in type I fibers, an increase in type IIx fibers, and smaller cross-sectional area of type I and IIa fibers in the gastrocnemius muscle have been reported [20]. On the other hand, several studies show that PAD results in an increase in the muscle area occupied by oxidative type I fibers [21–23] (reviewed in [24]). Whether specific fiber type alterations are associated with more severe mobility impairment in PAD patients is unknown.

The considerable variability in reports on fiber type alterations in PAD led us to hypothesize that fiber type composition in the gastrocnemius muscle would directly relate to walking performance in PAD. Additional features that may relate to functional performance were also evaluated, including capillary density and mitochondrial content. These analyses suggest potential new targets in muscle for the development of effective interventions to improve muscle function and mobility in PAD patients.

## Methods

### Participant identification, recruitment methods, and ethical approval

PAD (n = 26) and non-PAD (n = 7) participants were identified from among participants in five studies at Northwestern University Feinberg School of Medicine. Two studies were observational cohort studies [the walking and leg circulation study iii (WALCS III) and Biomarker Risk Assessment in Vulnerable Outpatients (BRAVO)] [25–27] and three studies were randomized trials [Group Oriented Arterial Leg Study (GOALS), Progenitor Cells to Improve Leg Functioning in PAD (PROPEL)] [28–30], and Resveratrol To Improve Outcomes in Older People with PAD (RESTORE). For the observational studies, participants from WALCS III and BRAVO were recruited from among consecutively identified PAD patients at Chicago-area medical centers who were evaluated in vascular surgery, cardiology, general medicine, endocrinology, and geriatric clinics [25–27]. Non-PAD participants were identified either among consecutive men and women age 65 and older without risk factors for PAD or cardiovascular disease in a large general internal medicine practice at Northwestern who were screened with the ankle brachial index (ABI) and found to have an ABI of 0.90 to 1.30 [25] or from potential study participants for the randomized trials who were found to have a normal ABI at the screening/baseline study visit [28–30].

### Inclusion and Exclusion Criteria

All PAD participants in these analyses had a baseline ABI value of <0.90. All non-PAD participants included in these analyses had a baseline ABI of 0.90 to 1.30.

Exclusion criteria have been reported [25–30] and consisted of severe functional limitation, characterized by inability to walk without a walker or wheelchair, significant cognitive impairment, terminal illness, recent major operation or recent major cardiovascular event and lack of willingness to return for the required study visits.

### Comorbidities

Presence of comorbid diseases and smoking history were obtained by administering questionnaires in a standardized fashion.

### Medications

Participants were asked to bring their medication bottles or a complete list of their medications to their baseline study visit. Names of each medication were recorded. A study investigator (MMM) reviewed each medication named and indicated whether each medication was a statin or not.

### Intermittent claudication

The presence and characteristics of exertional leg symptoms were assessed using the San Diego claudication questionnaire [31]. Participants with exertional calf pain that did not begin at rest and that resolved within 10 min of rest were classified with intermittent claudication [31].

### Ankle brachial index

The ankle brachial index (ABI) was measured with a handheld Doppler probe (Nicolet Vascular Pocket Dop II, Golden, CO) to measure systolic blood pressures after the participant rested supine for 5 min. Pressures were measured in the following order, and then repeated in reverse order: right brachial, dorsalis pedis, and posterior tibial arteries; left dorsalis pedis, posterior tibial, and brachial arteries. The ABI was calculated by dividing average pressures in each leg by the average of the four brachial pressures [32, 33].

### Six-min walk

Following standardized instructions to complete as many laps as possible, participants walked back and forth over a 100-foot hallway for 6 min [1, 2, 5, 34]. Participants were instructed to walk continuously with the goal of covering as much ground as possible within the 6 min. Participants were allowed to stop and rest if needed. A research assistant walked with and slightly behind the participant, so that the research assistant did not pace the participant. Standardized words of encouragement were given at 1-min intervals, for example, “One minute has passed. You’re doing well; keep up the good work.” The distance covered after 6 min and the distance at onset of leg symptoms were recorded.

### Four-meter walks

Four-meter walks were performed at “normal” and “fast” pace to measure walking velocity, based on previous studies [35, 36]. Each walk was performed twice and the faster walk in each pair was used.

### Muscle biopsies

A single investigator (RLS) collected all muscle biopsies from the medial head of the gastrocnemius muscle, at the point that was approximately 67 % of the distance between the medial malleolus and the medial aspect of the proximal tibia. Anesthesia was achieved with subcutaneous lidocaine. Subcutaneous and adipose tissue were dissected until muscle was identified. Approximately 100 mg of muscle tissue was mounted in trigacanth gum on cork and immediately frozen in liquid-nitrogen cooled isopentane to be processed for immunohistochemical analysis. The fascia was closed with absorbable suture, the wound was closed with subcuticular sutures, and the skin was closed with steri-strips.

### Histochemistry/Immunohistochemistry

Seven-micrometer sections of the gastrocnemius muscle were cut in a cryostat and allowed to dry at room temperature for 1 h. Slides were stored at −20 °C until processed as described below:

For fiber type determination (n = 26 PAD, n = 7 non-PAD), unfixed consecutive sections were incubated overnight at 4 °C with anti-laminin (#L9393; Sigma-Aldrich, St. Louis, MO, USA) and isoform-specific myosin heavy chain (MyHC) antibodies: MyHC type I (BA.D5; IgG2b), IIa (SC.71; IgG1) and IIx (6H1; IgM), all from Developmental Studies Hybridoma Bank (DSHB; Iowa City, IA, USA). The next day, slides were incubated with anti-rabbit IgG H+L AMCA (#C1-1000; Vector Laboratories, Inc., Burlingame, CA, USA) and immunoglobulin-specific secondary antibodies: goat anti-mouse IgG2b AF647 (#A21242), goat anti-mouse IgG1 AF488 (#A21121), and goat anti-mouse IgM AF555 (#A21426), all from Invitrogen (Grand Island, NT, USA), for 1 h. Slides were post-fixed in methanol for 5 min and then mounted (#H-1000; Vector Laboratories). The entire cross-section of approximately 1000 fibers (range 600–1600) per subject was analyzed for minimum feret diameter and fiber type. Minimum feret diameter was determined using an interactive automated analysis program in ZEN 2 (blue edition, v2.0, Zeiss, Oberkochen, Germany). The program calculated minimum fiber feret diameter based on the laminin staining, which outlines fibers. Each fiber was then assigned to either type I or type II based on the intensity of staining within the Cy5 (type I) or FITC and TRITC filters (type IIa and type IIx, respectively).

For lectin staining, to quantify capillary density, slides were blocked for 1 h in normal horse serum (#S-2012; Vector Laboratories), incubated in TRITC-labeled lectin (#L4889; Sigma-Aldrich) for 90 min at room temperature, and then mounted with fluorescent mounting media (Vectashield, #H-1000; Vector Laboratories). The lectin system from *Ulex europaeus* has been validated as an accurate vascular endothelial marker in human skeletal muscle [37, 38]. The lectin+ capillaries were counted and expressed per fiber. Due to the orientation and quality of the biopsy, quantification could only be performed on n = 18 PAD subjects. Approximately 600 fibers (range 400–750) per subject were in cross section to be accurately counted for lectin + staining.

For Oil Red O (ORO) staining, ORO stock [700 mg ORO (#O0625; Sigma-Aldrich) and 100 mL 60 % Triethyl phosphate (#538728; Sigma-Aldrich)] was filtered using Whatman® #1 filter paper then diluted 3:2 with double distilled water (ddH_2_O) to make the ORO working solution. Slides were fixed in 4 % paraformaldehyde for 3 min at room temperature then rinsed in ddH_2_O. Slides were then incubated in working ORO solution for 2 h, rinsed in ddH_2_O, and mounted with mounting media (#H-5501; Vector Laboratories).

For succinate dehydrogenase (SDH) activity staining, slides were incubated in 0.75 mM nitrotetrazolium blue (#N6876; Sigma-Aldrich), 125 mM succinic acid disodium (#224731; Sigma-Aldrich), and 75 mM PBS, pH 7.4, for 1 h at 37 °C. Following a series of acetone rinses, slides were mounted. Fibers that stained strongly with SDH (++) were counted as type I fibers, fibers that stained intermediate (+) were type IIa fibers, and those that did not stain for SDH (−) were type IIa/x hybrids. Cavities were evident in type I and IIa fibers, but not in IIa/x hybrids because of the lack of SDH staining in the latter. Fibers containing distinct cavities in SDH staining greater than 5 % of the fiber area were quantified and expressed as a percentage of total fibers. Due to the orientation and quality of the biopsy, quantification could only be performed on n = 18 PAD subjects. Approximately 1000 fibers (range 750–1300) were analyzed per subject.

For staining of mitochondrial complex I, subunit 20 and complex IV, subunit 1 (COX-1), slides were fixed in 4 % paraformaldehyde for 10 min, washed with TBST (1 % Tween-20), then blocked for 1 h in TBST with 1 % normal goat serum. Next, slides were incubated overnight at 4 °C in anti-NDUFB8 (complex I-20; #ab110242; Abcam, Cambridge, MA) and anti-MTCO1 (COX-1; #ab14705; Abcam) antibodies in TBST with 1 % normal goat serum. Following TBST washes, slides were incubated in biotin goat anti-mouse IgG1 (#115-065-205; Jackson Immuno Research, West Grove, PA, USA) and anti-mouse IgG2a AF488 (#A21121; Invitrogen) secondary antibodies in TBST with 1 % normal goat serum for 1 h. Slides were reacted with streptavidin-horseradish peroxidase included with the TSA kit (#T20935; Invitrogen), incubated in TSA AF594 (#T20950; Invitrogen) in amplification diluents for 15 min, then mounted (#H-1000; Vector Laboratories).

For staining of mitochondrial COX-1 and microtubule-associated protein light chain 3 (LC3), slides were fixed in 4 % paraformaldehyde for 10 min, washed with TBST (1 % Tween-20), then blocked for 1 h in TBST with 5 % normal goat serum. Slides were incubated overnight at 4 °C in anti-MTCO1 (cox-1; #ab14705; Abcam) and anti-LC3B (#NN100-2220; Novus Biologicals, Littleton, CO, USA) antibodies in TBST with 5 % normal goat serum. Following TBST washes, slides were incubated for 1 h in biotin goat anti-rabbit IgG (#111-065-003; Jackson Immuno Research) and anti-mouse IgG2a AF488 (#A21121; Invitrogen) secondary antibodies in TBST with 5 % normal goat serum. Lastly, slides were incubated in streptavidin AF594 (#S32356; Thermo Scientific, Waltham, MA, USA) for 15 min, then mounted (#H-1000; Vector Laboratories).

For staining of LC3 and lysosome associated membrane protein 2 (LAMP2), slides were fixed in ice-cold acetone for 10 min, washed with TBST (1× TBS with 1 % Tween-20), blocked for 1 h in TBST with 5 % normal goat serum, then incubated overnight at 4 °C in anti-LC3B (#NN100-2220; Novus Biologicals) and anti-LAMP2 (#ab25631; Abcam) antibodies in TBST (0.5 % Tween-20) with 5 % normal goat serum. Following TBST (1 % Tween-20) washes, slides were incubated for 1 h in biotin goat anti-rabbit IgG (#111-065-003; Jackson Immuno Research) in TBST (0.5 % Tween-20) with 5 % normal goat serum, then washed in TBST (1 % Tween-20). Next, slides were incubated for 2 h in streptavidin AF594 (#S32356; Thermo Scientific) and anti-mouse IgG2a AF488 (#A21121; Invitrogen) in TBS with 5 % normal goat serum. Slides were then washed in TBS and mounted (#H-1000; Vector Laboratories).

### Image acquisition and analysis

Images were captured at either ×10 or ×20 magnification at room temperature with a Zeiss upright microscope (AxioImager M1; Zeiss, Oberkochen, Germany) and analysis carried out using the Zeiss ZEN 2 software (blue edition, v2.0). To capture the entire muscle cross-section (200–400 fibers/subject), the tiles feature within Zen was utilized. Images of COX-1 and LC3 co-staining were also captured at ×60 magnification at room temperature with a Nikon AIR+ confocal imaging system and analyzed with NIS-Elements C Software (Nikon Instruments, Inc., Melville, NY, USA). Investigators were blinded to sample subject group for all analyses.

### Real-time quantitative PCR

RNA was extracted from PAD muscle biopsies using the RNeasy Mini Kit (Cat. No. 74104, Qiagen, Valencia, CA, USA). RNA quality and integrity were assessed using the Agilent 2100 Bioanalyzer. Because of low starting quantities, RNA of sufficient quality was obtained from 18 subjects. Reverse transcription was performed using the Superscript® VILO™ cDNA Synthesis Kit (Cat. No. 11754050, Life Technologies, Carlsbad, CA, USA). Quantitative real-time PCR was performed using SYBR Select Master Mix (Cat. No. 4472903, Life Technologies) and gene expression was normalized to the geometric mean of three housekeeping genes: β2 microglobulin, phosphoglycerate kinase, and 18S RNA. Primer pairs are presented in Additional file 1: Table S1.

### Statistical analyses

Differences in clinical and muscle characteristics between PAD and non-PAD participants were compared using Fisher’s exact testing for categorical variables and t-testing for continuous variables. Simple correlation coefficients between muscle fiber characteristics and functional performance measured by normal and fast paced 4-m walk velocity as well as six-min walking distance were estimated and the significance levels of nonzero correlations were obtained among PAD participants. The multiple linear regression analysis was performed to examine the association of the type I fiber size (quantified by the minimum feret diameter) and the proportion of type I fibers with functional performance. Furthermore, the interactions between the proportion and size of type I fiber are also tested in the multiple regression analysis. Associations were considered significant at P ≤ 0.05 based on a two-sided test and trends declared at P ≤ 0.10. All analyses were performed using SAS (Version 9.4, SAS Institute Inc., Cary, NC).

## Results

### Study participant characteristics

The demographic data and clinical characteristics of PAD and non-PAD subjects are shown in Table 1.

**Table 1:** Characteristics of non-PAD and PAD participants

Continuous variable	Non-PAD (n = 7)	PAD (n = 26)	*P* value
Age (year), mean (SD)	69.71 (4.99)	66.81 (10.20)	0.474
Ankle brachial index, mean (SD)	1.13 (0.10)	0.63 (0.14)	<0.0001
Body mass index (kg/m^2^)^a^, mean (SD)	25.15 (2.51)	28.41 (4.75)	0.257
Categorical variable			
Male sex, n	4	15	1.000
African Americans, n	1	11	0.223
Current smoker^a^, n	0	14	0.224
Angina^a^, n	0	2	1.000
Myocardial infarction^a^, n	0	2	1.000
Heart failure^a^, n	0	0	1.000
Stroke^a^, n	0	2	1.000
Pulmonary disease^a^, n	0	5	1.000
Cancer^a^, n	2	3	0.068
Diabetes^a^, n	0	9	0.532
Intermittent claudication^a^, n	0	5	1.000
Statin use^b^, n	2	15	1.000

^a^Based on n = 3 non-PAD

^b^Based on n = 4 non-PAD

### Fiber size, but not fiber type distribution, relates to walking performance in subjects with PAD

**Fig. 1:**
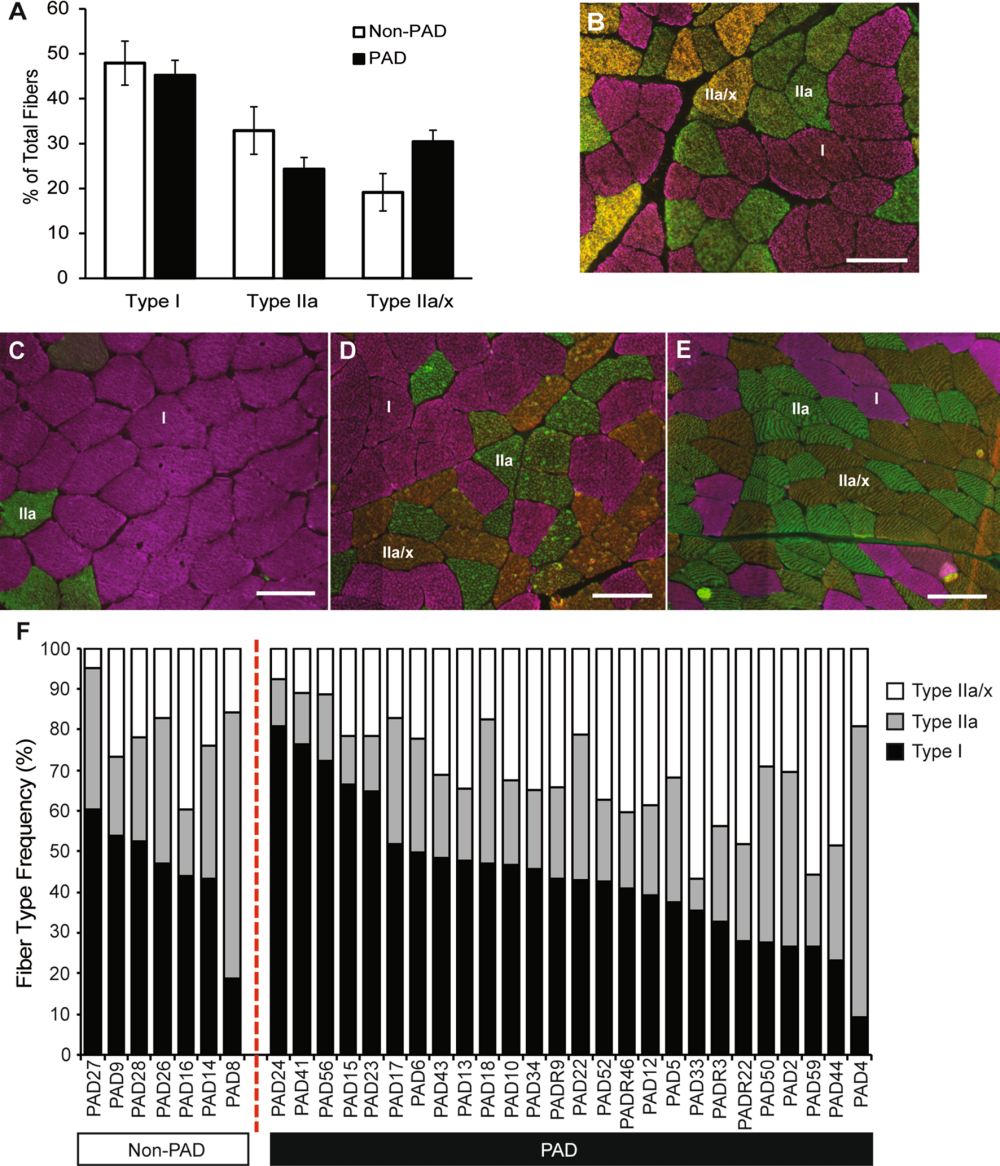
Fiber type analysis of gastrocnemius muscle sections using isoform-specific myosin heavy chain (MyHC) immunohistochemistry. **A** Quantification (mean ± SEM) of type I (*pink*), IIa (*green*), and IIa/x (*yellow*/*orange*) muscle fibers, shown in representative images, of non-PAD (**B**; n = 7) and PAD (**C**–**E**; n = 26) participants. **C**–**E**) Representative images of PAD subjects with primarily type I fibers, 50 % type I and 50 % Type II, and primarily type II fibers, respectively; **F** individual variation in fiber type distribution between non-PAD and PAD subjects (approximately 1000 fibers analyzed per subject). *Scale bar* = 100 µm

Quantifying the relative abundance of type I, IIa and IIx fibers showed that PAD participants exhibited trends toward greater mean frequency of hybrid type IIa/x fibers (P = 0.058) and reduced frequency of IIa fibers (P = 0.088), but had a similar mean frequency of type I fibers compared to non-PAD participants (Fig. 1A). Fibers expressing exclusively IIx MyHC were not detected. Non-PAD subjects exhibited a relatively even fiber type distribution (approximately 50 % type I and 50 % type II fibers; representative image, Fig. 1B), typical of healthy muscle, whereas PAD subjects demonstrated a wide heterogeneity of fiber type distributions. Representative images from PAD subjects with a higher percentage of type I fibers (Fig. 1C), an even distribution of type I and type II fibers (Fig. 1D), and a higher percentage of type II fibers (Fig. 1E) illustrate the variability. All individual subject fiber type distributions are shown in Fig. 1F and the relative distribution is shown in Additional file 2: Figure S1. Type I fiber frequency in individuals with PAD ranged from 9 to 81 % (19 to 60 % in non-PAD), type IIa abundance ranged from 8 to 72 % (20 to 65 % in non-PAD), and type IIa/x fibers ranged from 5 to 56 % (5 to 40 % in non-PAD).

We determined if fiber type frequency was associated with walking performance in PAD subjects, measured with 6-min walk distance, and normal- and fastest-paced 4-min walk velocities. Although the proportion of slow-twitch, type I fibers alone did not correlate with performance, type I fiber size, quantified by the minimum feret diameter, was strongly correlated to both normal- and fastest-paced 4-min walk velocity (normal-paced r = 0.4940, P = 0.010; fastest-paced r = 0.4944, P = 0.010, Table 2, Additional file 3: Figure S3A, B). The overall average minimum feret diameter was also positively correlated to both normal- and fastest paced 4-min walk velocity (r = 0.4804, P = 0.013 and r = 0.4137, P = 0.036, respectively, Additional file 3: Figure S3C, D). There was no correlation between fiber type or size and 6-min walk performance (Table 2). Further, there was a positive interaction effect between the type I fiber percentage and type I fiber size on the normal-paced 4-min walk velocity (P = 0.038).

**Table 2:** Correlations of functional measures with muscle characteristics in PAD patients (n = 26)

	Pearson correlation coefficients (Prob > |r| under H0: Rho = 0, number of observations)					
	6-min walk distance (m)		4-min walking velocity, normal-paced (m/s)		4-min walking velocity, fastest-paced (m/s)	
	r value	P value	r value	P value	r value	P value
% Type I	0.0067	0.975	0.1422	0.488	0.0785	0.703
% Type IIa	−0.0539	0.798	−0.2054	0.314	−0.2433	0.231
% Type IIa/IIx	0.0456	0.829	0.0210	0.919	0.1435	0.485
Min. feret, Type I	0.3175	0.122	*0.4940*	*0.010*	*0.4944*	*0.010*
Min. feret, Type II	0.2676	0.196	*0.4317*	*0.028*	0.3713	0.062
Min. feret, average	0.3121	0.129	*0.4804*	*0.013*	*0.4137*	*0.036*
Lectin/fiber^a^	0.0537	0.813	−0.0208	0.925	−0.0783	0.723
% Fibers with cavities in SDH^a^	−0.1991	0.460	0.4426	0.086	0.0993	0.714
*PGC1α* mRNA^a^	−0.1025	0.686	0.0832	0.743	0.1580	0.531
*CREB* mRNA^a^	0.0750	0.768	−0.2533	0.310	−0.2675	0.283
*CTRC1* mRNA^a^	0.0431	0.871	−0.2346	0.349	−0.2495	0.318
*HIF1α* mRNA^a^	0.2072	0.409	−0.2480	0.321	−0.1937	0.441

Italicized text indicates significance (P < 0.05)

^a^n = 18

### Maintenance of capillary density in PAD

Consistent with previous reports [20, 24], there was a trend for PAD subjects to have a greater number of capillaries/fiber than non-PAD (1.15 ± 0.28 vs. 1.72 ± 0.13 for non-PAD and PAD subjects, respectively; P = 0.082; Additional file 4: Figure S4, representative images shown in A and B, quantified in C). However, capillary density did not correlate to fiber type (r = −0.1023 P = 0.642), fiber minimum feret diameter (r = 0.1396, P = 0.525), nor to function (Table 2).

### Mitochondrial oxidative activity is impaired in PAD

**Fig. 2:**
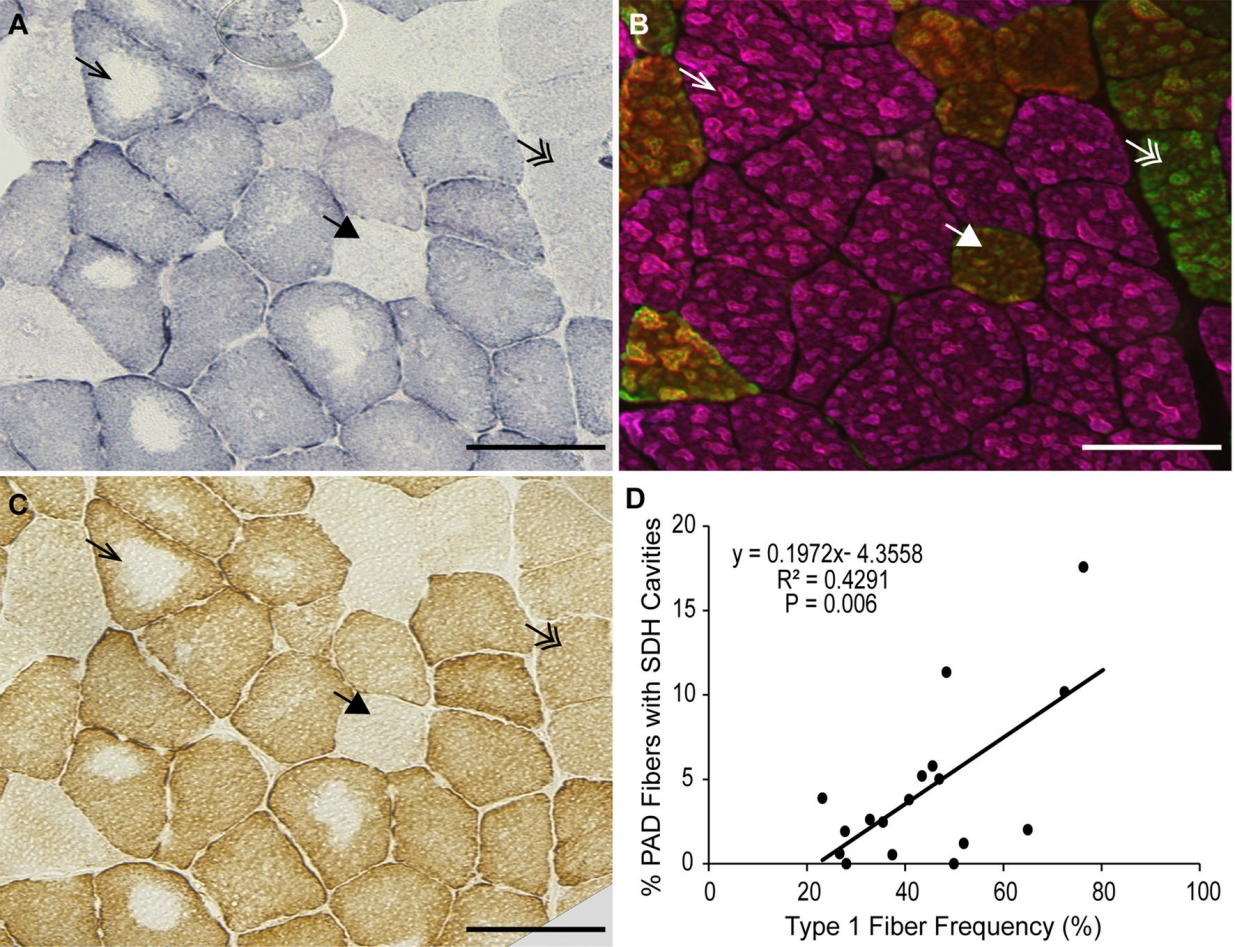
Representative images of serial PAD gastrocnemius muscle sections stained for mitochondrial activity and fiber type. **A** succinate dehydrogenase (SDH) activity; **B** myosin heavy chain (MyHC) composition by immunohistochemistry; **C** cytochrome *c* oxidase activity. *Arrows* point to the same fiber in serial sections. *Arrow*, type I fiber (*pink*); *double arrow*, type IIa fiber (*green*); *large arrowhead*, type IIa/x hybrid fiber (*yellow*/*orange*). **D** Correlation between the percent of total fibers with SDH cavities and type I fiber frequency in PAD subjects (n = 18, approximately 1000 fibers counted per subject). *Scale bar* = 100 µm

Muscle sections were stained for succinate dehydrogenase (SDH) activity (Fig. 2A) to evaluate mitochondrial oxidative capacity that was then compared to fiber type (Fig. 2B) on serial sections. As expected, fibers that stained strongly for SDH activity (++) were type I slow-twitch fibers, fibers that stained intermediate for SDH (+) were type IIa fibers, and fibers with lowest SDH staining (−) were type IIa/x hybrids.

**Fig. 3:**
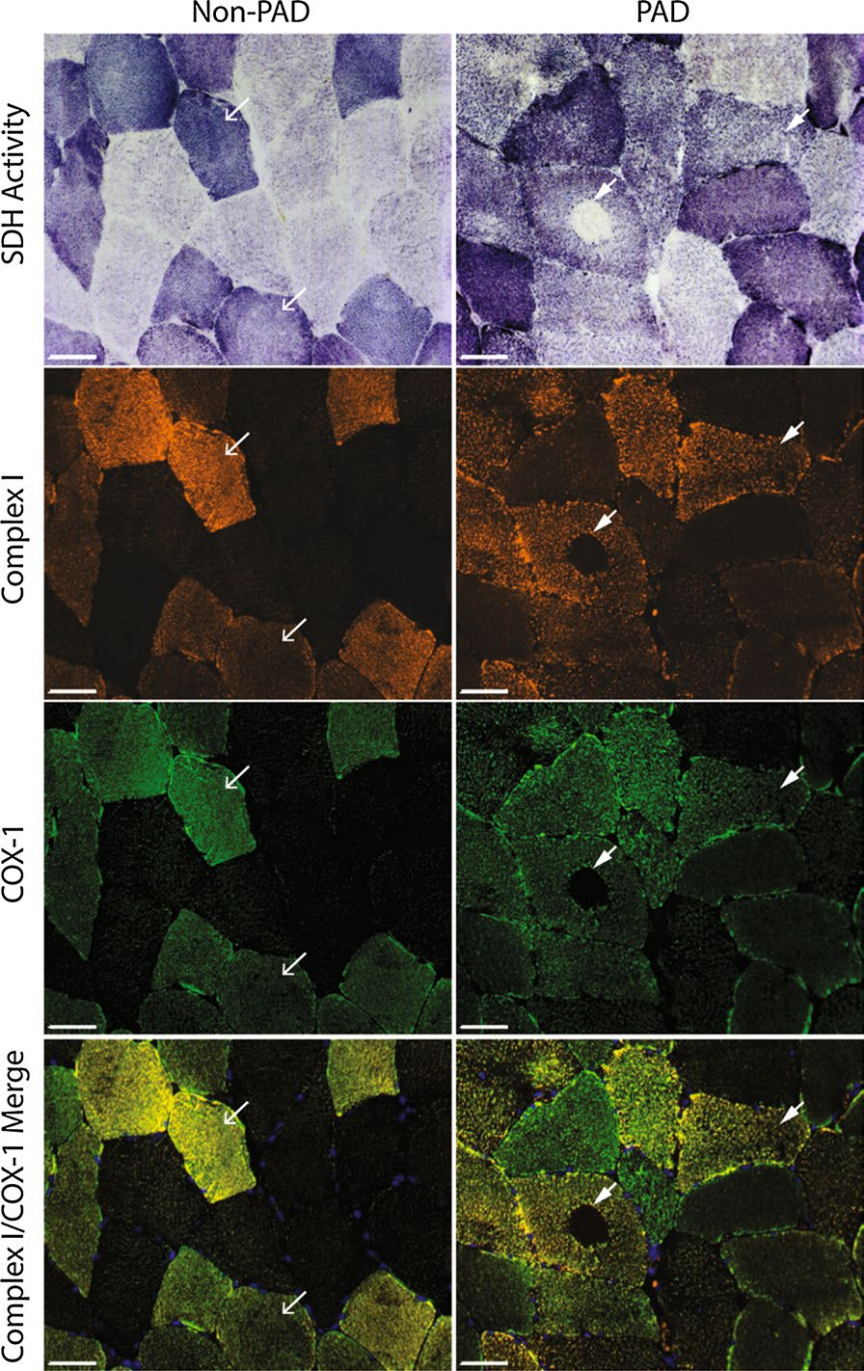
Representative images of non-PAD and PAD gastrocnemius muscle sections stained for mitochondrial activity and mitochondrial electron transport proteins. *Top row* shows succinate dehydrogenase (SDH) activity (dark fibers are type I, intermediate fibers are type IIa, and light fibers are type IIa/x), *second row* shows mitochondrial complex I, subunit 20 (*orange*), *third row* shows mitochondrial complex IV, subunit I (COX-1; *green*) staining and *bottom row* shows complex I and COX-1 merged. *Arrows* within the same column point to same fibers. *Scale bar* = 50 µm

SDH activity staining indicates the presence of both intermyofibrillar and subsarcolemmal mitochondrial activity; PAD participants showed a loss of intermyofibrillar SDH activity in the center of up to 17 % of their muscle fibers (4.61 ± 4.76 %, mean ± SD; Fig. 2A). These cavities were also devoid of cytochrome *c* oxidase (complex IV; COX) activity (Fig. 2C), but were not centrally necrotic, as evidenced by the presence of MyHC staining in the center of the fibers (Fig. 2B). Additionally, the fibers showed no accumulation of ectopic lipid in the center (ORO staining; Additional file 5: Figure S5). The percentage of fibers with absence of SDH activity in the center (cavities) was positively correlated with type I fiber frequency (r = 0.6499; P = 0.006; Fig. 2D). Immunohistochemical analysis showed that in PAD subjects, proteins comprising complexes I and IV (COX-1) of the electron transport system were undetectable in the center of fibers that lacked SDH activity (Fig. 3, right column), indicating that loss of SDH activity is due to the absence of intermyofibrillar mitochondria. The absent intermyofibrillar mitochondria were not evident in non-PAD muscle samples (Fig. 3, left column).

**Fig. 4:**
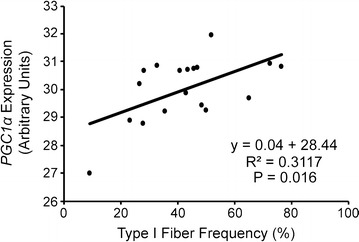
Correlation between mRNA levels of *PGC1α* and type I fiber frequency in PAD gastrocnemius muscle samples (n = 18, approximately 1000 fibers counted per subject to quantify type I fiber frequency)

We analyzed PAD gastrocnemius muscle homogenate for mRNA levels of peroxisome proliferator-activated receptor gamma coactivator 1α (PGC1α), the master regulator of mitochondrial biogenesis, as well as additional genes important during mitochondrial biogenesis, cAMP response element-binding protein (CREB) and CREB-regulated transcription coactivator 1 (CRTC1). Additionally, hypoxia-inducible factor 1α (HIF1α) expression, which has been shown to stimulate mitochondrial biogenesis following short-term hypoxia, was evaluated. Within PAD subjects, *PGC1α* mRNA correlated positively with the percentage of type I fibers (r = 0.5580, P = 0.016; Fig. 4), as would be expected in normal muscle. No correlation between *PGC1α, CREB, CRTC1,* nor *HIF1α* expression and the percentage of fibers showing absence of SDH staining was apparent (Additional file 6: Figure S6). Further, expression of *PGC1α, CREB, CTRC1, and HIF1α* did not correlate with any functional performance measures in PAD subjects (Table 2).

### Autophagic accumulation in the center of PAD fibers

**Fig. 5:**
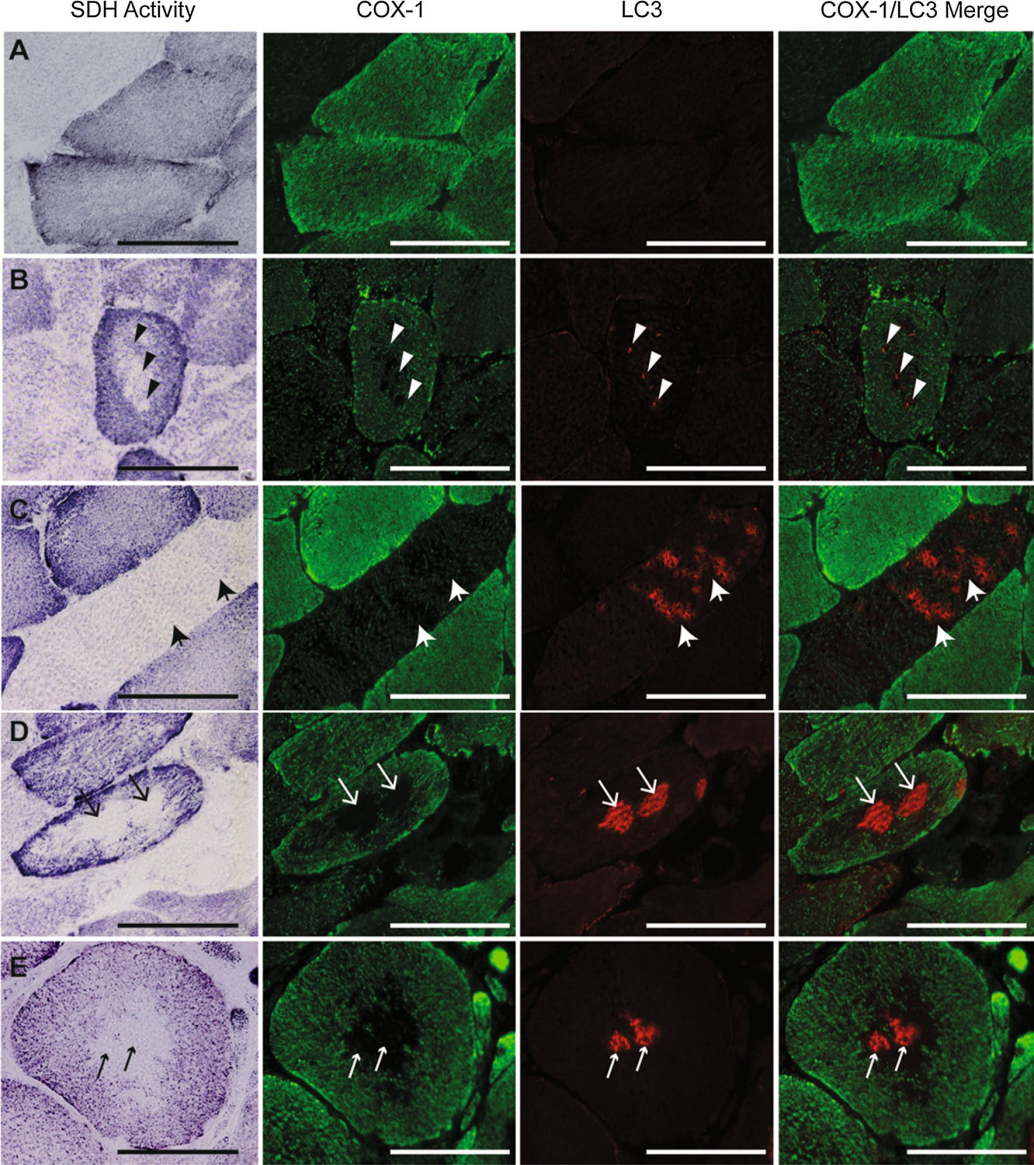
Representative images of the variation of LC3 staining associated with cavities in SDH activity and mitochondrial COX-1 staining in PAD gastrocnemius muscle sections. *First column* shows succinate dehydrogenase (SDH) activity (*dark fibers* are type I, intermediate fibers are type IIa, and light fibers are type IIa/x), *second column* shows mitochondrial COX-1 protein (*green*), third column shows LC3 (*red*) and *fourth column* shows COX-1 and LC3 merged. Examples of normal fibers with no LC3 staining or COX-1 cavities (*row* **A**), punctate LC3 staining where a COX-1 cavitiy is forming (row **B**), elevated LC3 staining with low COX-1 staining (*row* **C**), LC3 accumulation in areas lacking COX-1 staining (row **D**), and LC3 plaque in the center of an SDH/COX1 cavity (COX-1;* row* **E**). *Arrows* within the *same row* point to areas of LC3 accumulation in same fiber. *Scale bar* = 100 µM

**Fig. 6:**
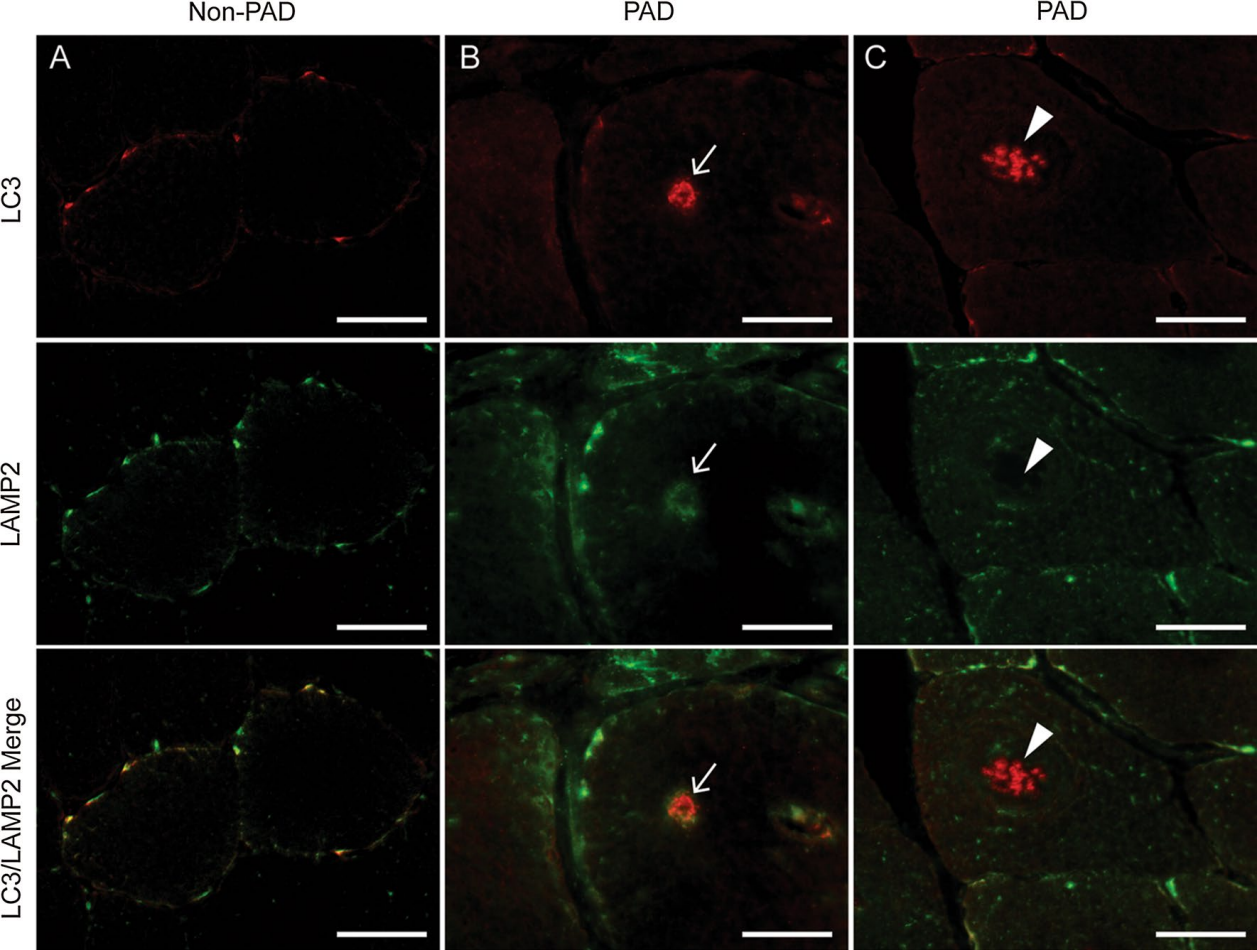
Representative images of gastrocnemius muscle sections from non-PAD and PAD subjects stained for LC3, an autophagosome marker (*red*) and LAMP2, a lysosome marker (*green*). **A** In non-PAD, very little LC3 and LAMP2 staining is apparent. In PAD, rare fibers show co-localization of accumulated LC3 and LAMP2 (**B**), whereas the majority of fibers have elevated LC3 accumulation but no co-localization with LAMP2 (**C**). *Arrows* in the same column point to the same areas of LC3 accumulation in the center of the fiber with or without LAMP2 co-localization. *Scale bar* = 50μm

Muscle sections were co-stained with antibodies against COX-1 and LC3 protein, the latter being an autophagosome marker (Fig. 5). Fibers with normal mitochondrial activity in both PAD and non-PAD subjects showed ubiquitous COX-1 staining throughout the fiber, indicative of intermyofibrillar mitochondria, and stronger staining around the periphery, where subsarcolemmal mitochondria are localized. In normal muscle fibers, both SDH++/COX-1 high and low muscle fibers demonstrate very little detectable LC3 staining, indicating relatively low levels of autophagy (Fig. 5, row A). In PAD, fibers showing small central regions with absent mitochondrial protein and activity exhibited punctate LC3 staining localized to those regions (Fig. 5, row B). However, other PAD fibers exhibited varying degrees of LC3 accumulation in the center with low or absent SDH and COX-1 staining, suggesting autophagy may be increased in subjects with PAD (Fig. 5, rows C, D). Overall, fibers that lacked COX-1 protein and SDH activity in their cores demonstrated clear accumulation of LC3 staining (Fig. 5, row E). The loss of the mitochondrial network in the center of fibers with LC3 accumulation can be seen more clearly with confocal imaging, presented in Additional file 7: Figure S7. Some fibers had relatively diffuse LC3 staining within the SDH/COX-1 cavities (Additional file 7: Figure S7A), while the larger, more fully developed cavities contained a dense LC3 plaque in the center (Additional file 7: Figure S7B). To determine if the remaining LC3 staining was due to improper fusion of the autophagosome and the lysosome, muscle samples were co-stained with LC3 and LAMP2, a lysosome membrane protein (Fig. 6). In non-PAD, diffuse, weak LC3 and LAMP2 staining was visible within the fibers (Fig. 6a). In PAD subjects, rare fibers showed overlap of LC3 and LAMP2 (Fig. 6b), whereas the majority of fibers showed LC3 accumulation in the center of the fiber but no clear overlap with LAMP2 (Fig. 6c).

### Fiber size may affect the degree of mitochondrial dysfunction

**Fig. 7:**
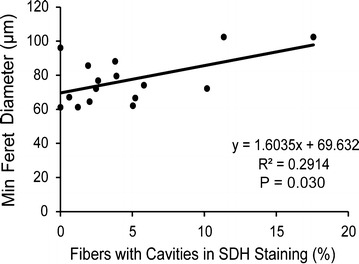
Correlation between average minimum fiber diameter and succinate dehydrogenase (SDH) cavities in PAD gastrocnemius samples. SDH cavities are quantified by the percentage of fibers with cavities in SDH activity staining

While capillary density did not correlate to fiber size (r = 0.1396, P = 0.525) or the percentage of fibers showing cavities in SDH staining (r = −0.0549, P = 0.845), the average minimum feret diameter positively correlated to the percentage of fibers with SDH cavities (r = 0.5398, P = 0.030; Fig. 7).

## Discussion

A novel finding of this study was the correlation between gastrocnemius muscle fiber size (minimum fiber diameter) and 4-min walking velocities in PAD subjects. While previous reports have indicated fiber cross sectional area decreases as much as 40 % with advancing PAD [20, 39], the current study is the first to show a positive correlation between fiber size and functional measures. In contrast, size was not correlated to 6-min walk distance, a measure of walking endurance. It is possible that in PAD patients, ischemia-related pain in the limb muscles causes patients to stop walking during the 6-min walk test, which may be less dependent on the muscle properties studied here.

A wide range of fiber type distributions was evident in PAD subjects in the current study, which was not correlated to walking performance or ABI, the latter being an accepted measure of disease severity in PAD [2, 4, 40, 41]. Although we initially hypothesized that those with the highest proportion of type I fibers may have the highest endurance, this was not the case, which may be due to preferential loss of intermyofibrillar mitochondria in type I fibers in PAD. Research has indicated there is a switch from a type II to a type I phenotype in the gastrocnemius muscle with advancing PAD [39]. We hypothesize that an overall decrease in fiber size combined with an increase in type I fiber area and concomitant mitochondrial loss in the center of oxidative fibers may, in combination, contribute to progressive mobility loss in PAD. This hypothesis warrants further investigation.

Previous work demonstrated impaired mitochondrial respiration and enhanced oxidative stress and damage in the muscles of PAD subjects [10, 24, 42]. Pipinos, et al. [10] reported reduced activity of complexes I, III, and IV of the electron transport system in gastrocnemius homogenates, but no difference in complex II activity. Complex II is the only complex of the electron transport system that is fully encoded by the nuclear genome, while the remaining 3 complexes contain mitochondrial-encoded subunits [43], suggesting impairment of the mitochondrial genome in PAD. In the current study, we showed a disruption of both succinate dehydrogenase (SDH, complex II) and cytochrome *c* oxidase (complex IV; COX) activities in the center of fibers of PAD subjects, which is not typically seen in healthy muscle fibers. Further, both the nuclear-encoded subunit 20 of complex I and the mitochondrial-encoded subunit 1 of complex IV were absent in the center of a significant number of fibers of PAD subjects, indicating absence of intermyofibrillar mitochondrial populations in the core of the fibers. Mitochondrial absence could be due to limited O_2_ and nutrient delivery to the center of the fiber, as is seen in canine models of heart failure [44]. This idea is further supported by the positive correlation between fiber size and the percentage of fibers lacking central mitochondria in the current study. However, recent reports have described succinate accumulation in various tissues (heart, liver, brain, and kidney) following ischemia and implicated the metabolite as a potential driver of reperfusion reactive oxygen species production [45]. Impaired SDH activity and the potential accumulation of succinate during repeated bouts of ischemia and reperfusion could be compounding factors leading to oxidative damage and impaired functional performance in PAD patients; these findings require further investigation.

The maintenance of healthy cellular energetics relies on a dynamic process of balancing the removal of damaged or dysfunctional mitochondria through mitochondrial-specific autophagy, known as mitophagy, and mitochondrial biogenesis [46]. In the current study, *PGC1α*, the master regulator of mitochondrial biogenesis, was highest in subjects with a greater percentage of oxidative fibers, consistent with normal muscle. No correlation between *PGC1α, CREB1, CRTC1,* and *HIF1α* expression and the percentage of fibers with depleted central SDH activity suggests impaired mitochondrial biogenesis was not a major contributor to mitochondrial loss. These data, combined with previous reports of mitochondrial dysfunction in PAD [47], led us to hypothesize the mitochondrial cavities in the core of some fibers of PAD subjects were due to elevated mitophagy and dysfunctional clearance of damaged mitochondria, as opposed to compromised biogenesis. One might expect an overcompensation of *PCG1α* to maintain mitochondrial density in the face of elevated mitophagy. However, the autophagosome debris in fibers lacking central mitochondria may have prevented healthy mitochondria from infiltrating the center of the fiber. On the other hand, the lack of correlation between mitochondrial biogenesis gene expression and SDH cavities may suggest that PAD fibers are not able to sufficiently increase mitochondrial biogenesis to maintain oxidative capacity.

The autophagic marker, LC3, was accumulated in the center of fibers that may be undergoing an elevated rate of mitophagy compared to healthy fibers. A consequence of aging is an impaired ability to degrade damaged mitochondria [48]. However, age was not associated with any skeletal muscle measure in our analyses. Still, there may be a similar mechanism in PAD subjects that prevents the proper removal of dysfunctional mitochondria. An increase in autophagosomes in the center of fibers in which mitochondria are actively being degraded may imply greater mitophagy; however, the LC3 plaque evident in some fibers in which mitochondria were clearly absent may indicate impaired fusion with the lysosome and incomplete recycling as is evident in other diseases, such as sporadic inclusion-body myositis [49]. This is further supported by incomplete co-localization of LC3 with the lysosome marker, LAMP2, in PAD fibers in the current study. It is important to note that the muscle samples collected capture a snapshot of a dynamic and ongoing process. There may be a progression within individual fibers of mitochondrial depolarization and death that may or may not successfully fuse with the autophagosome and, subsequently, the lysosome to be properly removed and recycled.

While the absence of mitochondrial activity within the center of myofibers has been reported in diseases such as central core disease (CCD), denervation, and hypertrophic cardiomyopathy [50], the mitochondrial disruption in the fibers of PAD appears distinct. CCD fiber morphology presents the most similarity to our findings but is caused by a genetic mutation in *RYR1*, the gene that encodes the skeletal muscle ryanodine receptor, disrupting calcium handling within the muscle. CCD is often accompanied by muscle weakness, delayed motor development, and orthopedic impairment (hip dislocation, scoliosis), and tends to present at a young age [51]. Denervation can result in a phenomenon known as “target fibers”, in which ringing of oxidative enzyme activity occurs within the center of myofibers, but a clear three-zone architecture is apparent [52], unlike the distinct cavities seen in PAD fibers. In hypertrophic cardiomyopathy, only type I fibers are affected and present with very small cavities at varying locations within the fiber [53], whereas both type I and IIa fibers in PAD participants presented with abnormal mitochondrial activity which were nearly always centrally located. Thus, we hypothesize that the chronic ischemic environment in PAD contributes to loss of intermyofibrillar mitochondria unique to this disease.

Although we hypothesize that poor 4-min walking performance in PAD is related to elevated mitophagy and impaired clearance of damaged mitochondria, an alternative conclusion is that mechanical capacity, rather than bioenergetics, may be limiting normal function in this diseased population. This is supported by our finding that myofiber size was positively correlated to 4-min walking performance in the current study. Research has indicated that low-impact, endurance exercise training improves walking performance in PAD but these studies did not evaluate fiber size adaptations, nor the relationship between fiber size and functional performance [54]. The pathology associated with PAD is likely multifaceted, but defects in the structure and function of the muscle, in addition to the vasculature, must be addressed in order to maximally improve function.

The most important limitation of this study is the relatively small sample size. Analysis of a greater number of patients with PAD would confirm the association between muscle morphology and function. Additionally, the duration of PAD was unknown in the subjects studied, which did not allow us to quantify the exposure to ischemia and relate them to muscle characteristics. Previous study demonstrates that the exact onset of PAD is not typically precisely identified [55]. Repeat biopsies several months apart, as well as comparison of the affected versus non-affected leg in PAD subjects, will contribute to our knowledge of the pathological progression of PAD in the gastrocnemius muscle. Lastly, it was assumed that the biopsy is representative of the entire muscle but the authors recognize that regions of localized damage may not accurately reflect the morphology of the entire muscle. Further, findings in one muscle may not reflect what is happening in other lower extremity muscles.

## Conclusions

In summary, among people with PAD, muscle fiber size was positively correlated to 4-min walk performance, and may be associated with the degree of mitochondrial dysfunction evident in PAD subjects. We speculate that the lack correlation of type I fiber frequency and function may be due to impaired mitochondrial activity within the center of muscle fibers, consequent to increased mitophagy in patients with PAD. Considering the morphological features of muscle may aide in the management plan for PAD patients to improve their overall quality of life.

## Additional files

10.1186/s12967-016-1030-6 Sequence of PCR primers used for real time quantitative PCR.
10.1186/s12967-016-1030-6 Whisker plot of the fiber type analysis of gastrocnemius muscle using isoform-specific myosin heavy chain (MyHC) immunohistochemistry. The distribution of type I, type IIa, and type IIa/x fibers is shown for non-PAD (n=7) and PAD (n=26) subjects. Approximately 1000 fibers were analyzed per subject.
10.1186/s12967-016-1030-6 Correlations between minimum feret diameter of gastrocnemius fibers from PAD subjects and walking performance (n=26, approximately 1000 fibers per subject). A) type I fiber minimum feret diameter versus normal-paced 4-m walking velocity; B) type I fiber minimum feret diameter versus fastest-paced 4-m walking velocity; C) average fiber minimum feret diameter versus normal-paced 4-m walking velocity; and D) average fiber minimum feret diameter versus fastest-paced 4-m walking velocity.
10.1186/s12967-016-1030-6 Lectin staining to quantify capillary density. Representative images of lectin binding to endothelial cells in non-PAD (A) and PAD (B) gastrocnemius muscle sections. C) Quantification of lectin staining, expressed as lectin+ capillaries/fiber in PAD (n=18) compared to non-PAD (n=7) participants (ANOVA, P=0.082). Approximately 600 fibers were analyzed per subject. Data represented as Mean ± SEM. Scale bar = 50 µm.
10.1186/s12967-016-1030-6 Representative images of oil red O (ORO, A) and succinate dehydrogenase activity (SDH, B) staining of gastrocnemius serial sections. Excessive lipid does not accumulate in the SDH cavities. Arrows point to same fibers. Scale bar = 100 µM.
10.1186/s12967-016-1030-6 Correlations between the percentage of fibers with cavities in SDH staining in PAD gastrocnemius muscle sections (n=18, approximately 1000 fibers analyzed per subject) and *PGC1α* (A), *CREB* (B), *CRTC1* (C), and *HIF1α* (D) mRNA expression.
10.1186/s12967-016-1030-6 Representative confocal images of PAD gastrocnemius muscle sections. A) LC3 (red) is diffuse throughout cavity where mitochondrial cytochrome *c* oxidase protein (complex IV; COX-1; green) is absent. B) LC3 has formed a plaque in the center of the COX-1 protein cavity. Scale bar = 10 µM.

## Abbreviations

ABI: ankle brachial index
CCD: central core disease
COX: cytochrome *c* oxidase
CREB: cAMP response element-binding protein 1
CRTC1: CREB-regulated transcription coactivator 1
HIF1α: hypoxia-inducible factor 1α
LAMP2: lysosomal associated membrane protein 2
LC3: microtubule-associated protein light chain 3
MyHC: myosin heavy chain
ORO: oil red O
PAD: peripheral artery disease
PGC1α: peroxisome proliferator-activated receptor gamma coactivator 1α
RYR1: ryanodine receptor 1
SDH: succinate dehydrogenase
