# Erratum to: Walking performance is positively correlated to calf muscle fiber size in peripheral artery disease subjects, but fibers show aberrant mitophagy: an observational study

**DOI:** 10.1186/s12967-017-1127-6

**Published:** 2017-02-23

**Authors:** Sarah H. White, Mary M. McDermott, Robert L. Sufit, Kate Kosmac, Alex W. Bugg, Marta Gonzalez-Freire, Luigi Ferrucci, Lu Tian, Lihui Zhao, Ying Gao, Melina R. Kibbe, Michael H. Criqui, Christiaan Leeuwenburgh, Charlotte A. Peterson

**Affiliations:** 1 College of Health Sciences and Center for Muscle Biology, University of Kentucky, 900 S Limestone CTW105, Lexington, KY 40536 USA; 2 Division of General Internal Medicine, Department of Medicine, Northwestern University Feinberg School of Medicine, 750 North Lake Shore Drive, 10th Floor, Chicago, 60611 USA; 3 Department of Preventive Medicine, Northwestern University Feinberg School of Medicine, Chicago, IL USA; 4 Department of Neurology, Northwestern University Feinberg School of Medicine, Chicago, IL USA; 5 National Institute on Aging, Baltimore, MD USA; 6 Department of Health Research & Policy, Stanford University, Stanford, CA USA; 7 Department of Surgery, Northwestern University Feinberg School of Medicine, Chicago, IL USA; 8 Jesse Brown Veterans Affairs Medical Center, Chicago, IL USA; 9 Department of Family Medicine and Public Health, University of California at San Diego, La Jolla, CA USA; 10 Department of Aging and Geriatric Research, University of Florida Institute on Aging, Gainesville, FL USA

## Erratum to: J Transl Med (2016) 14:284 DOI: 10.1186/s12967-016-1030-6

The original publication [1] contains incorrect references. Reference 15 should contain the following information: “Pipinos, II, Judge AR, Selsby JT, Shen Z, Swanson SA, Nella AA, Dodd SL. The myopathy of peripheral arterial occlusive disease: Part 1. Functional and histomorphological changes and evidence for mitochondrial dysfunction. Vasc Endovasc Surg. 2008;41:481–9.”

Reference 40 should contain the following information: “Ankle Brachial Index Collaboration, Fowkes FG, Murray GD, Butcher I, Heald CL, Lee RJ, Chambless LE, Folsom AR, Hirsch AT, Dramaix M, et al. Ankle brachial index combined with Framingham Risk Score to predict cardiovascular events and mortality: a meta-analysis. JAMA. 2008;300:197–208.”

The sentence “Low mitochondrial activity, measured either by respirometry from muscle biopsy specimens or using magnetic resonance spectroscopy, is associated with poorer treadmill walking in people with PAD” should refer to the references 11 and 14.
