# Supplementary material for: Walking performance is positively correlated to calf muscle fiber size in peripheral artery disease subjects, but fibers show aberrant mitophagy: an observational study
Source: J Transl Med. 2016 Sep 29;14:284. doi: 10.1186/s12967-016-1030-6 (PMC5043620; doi:10.1186/s12967-016-1030-6)
Supplement: Supplementary file 1 — 10.1186/s12967-016-1030-6 Sequence of PCR primers used for real time quantitative PCR. [file 12967_2016_1030_MOESM1_ESM.docx]

Supplemental Table 1. Sequence of PCR primers used for real time quantitative PCR.

| Gene* | Forward Primer | Reverse Primer |
| --- | --- | --- |
| 18S | 5’-TTCGAACGTCTGCCCTATCAA-3' | 5’-ATGGTAGGCACGGCGACTA-3’ |
| B2M | 5’-GATGAGTATGCCTGCCGTGT-3’ | 5’-TGCGGCATCTTCAAACCTCC-3’ |
| CREB1 | 5’-AACCAGCAGAGTGGAGATGC-3’ | 5’-CTGCTGGCATAGATACCTGGG-3’ |
| CTRC1 (TORC1) | 5’-CCAACATCATCCTCACAGTGACAG-3’ | 5’-AACTGGCTGTCGGAGTCAA-3’ |
| HIF1α | 5’-TCTTGGAAACGTGTAAAAGGATGC-3’ | 5’-CAGTCTACATGCTAAATCAGAGGGT-3’ |
| PGC1α | 5’-TCTGAGTCTGTATGGAGTGACAT-3’ | 5’-CCAAGTCGTTCACATCTAGTTCA-3’ |
| PGK1 | 5’-GTTGACCGAATCACCGACCT-3’ | 5’-GTCGACTCTCATAACGACCCG-3’ |

*B2M, β2 microglobulin; CREB1, cAMP response element-binding protein 1; CRTC1, CREB-regulated transcription coactivator 1; HIF1α, hypoxia-inducible factor 1α; PCG1α, peroxisome proliferator-activated receptor gamma coactivator 1α; PGK1, phosphoglycerate kinase.
