# Supplementary figures and images for: Walking performance is positively correlated to calf muscle fiber size in peripheral artery disease subjects, but fibers show aberrant mitophagy: an observational study
Source: J Transl Med. 2016 Sep 29;14:284. doi: 10.1186/s12967-016-1030-6 (PMC5043620; doi:10.1186/s12967-016-1030-6)

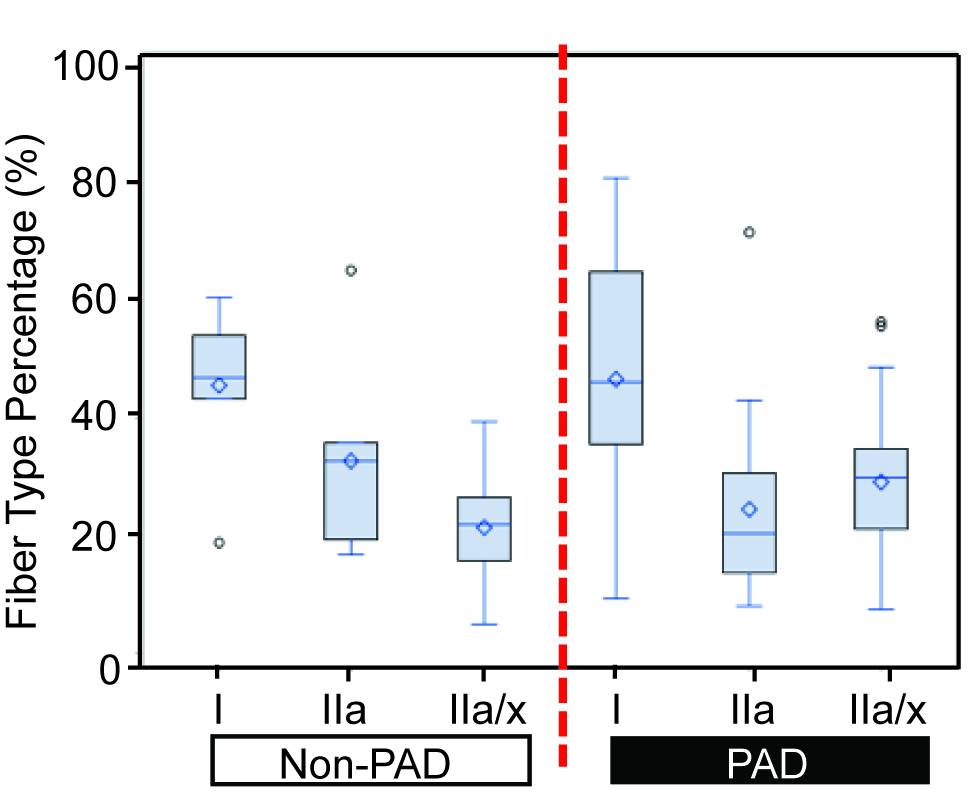

Supplement: Supplementary file 2 — 10.1186/s12967-016-1030-6 Whisker plot of the fiber type analysis of gastrocnemius muscle using isoform-specific myosin heavy chain (MyHC) immunohistochemistry. The distribution of type I, type IIa, and type IIa/x fibers is shown for non-PAD (n=7) and PAD (n=26) subjects. Approximately 1000 fibers were analyzed per subject. [file 12967_2016_1030_MOESM2_ESM.tif]

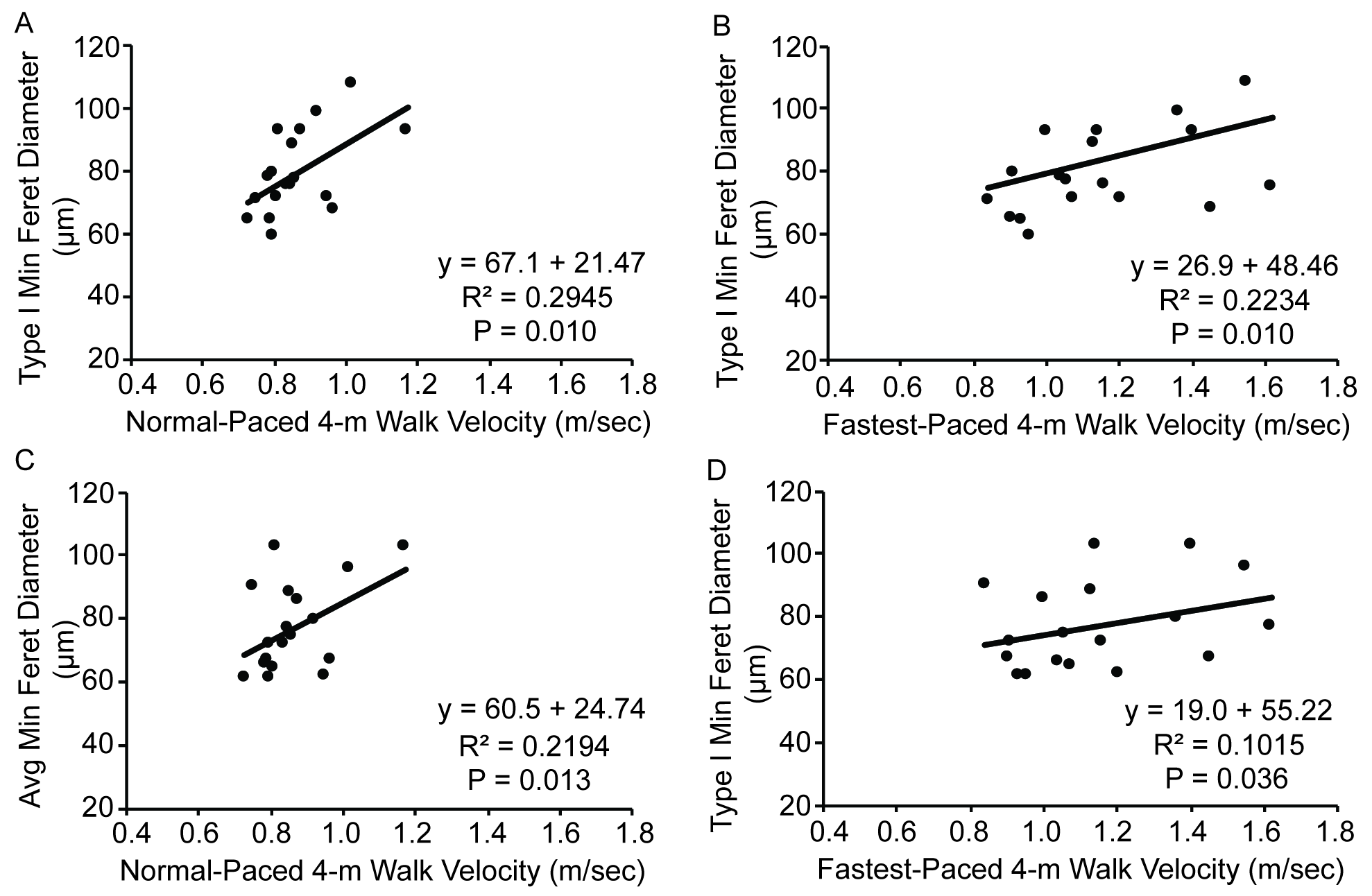

Supplement: Supplementary file 3 — 10.1186/s12967-016-1030-6 Correlations between minimum feret diameter of gastrocnemius fibers from PAD subjects and walking performance (n=26, approximately 1000 fibers per subject). A) type I fiber minimum feret diameter versus normal-paced 4-m walking velocity; B) type I fiber minimum feret diameter versus fastest-paced 4-m walking velocity; C) average fiber minimum feret diameter versus normal-paced 4-m walking velocity; and D) average fiber minimum feret diameter versus fastest-paced 4-m walking velocity. [file 12967_2016_1030_MOESM3_ESM.tif]

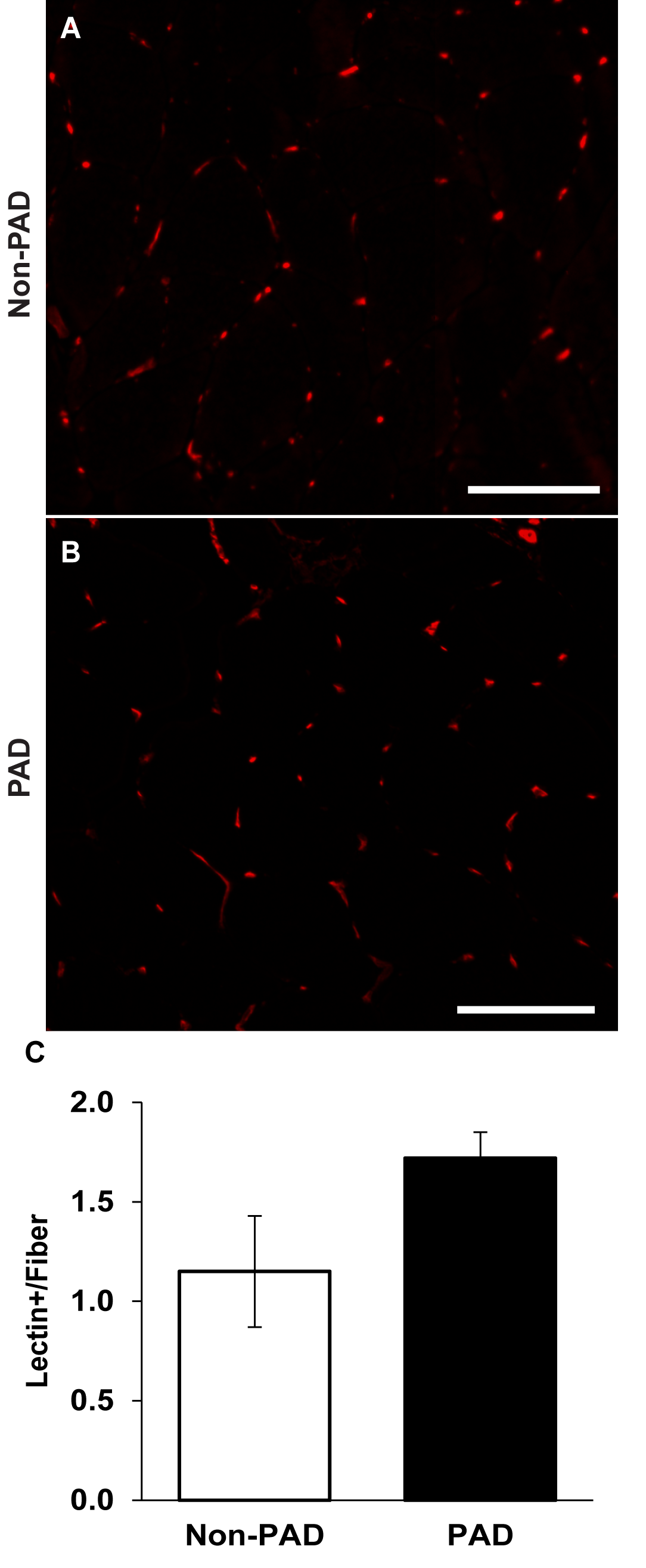

Supplement: Supplementary file 4 — 10.1186/s12967-016-1030-6 Lectin staining to quantify capillary density. Representative images of lectin binding to endothelial cells in non-PAD (A) and PAD (B) gastrocnemius muscle sections. C) Quantification of lectin staining, expressed as lectin+ capillaries/fiber in PAD (n=18) compared to non-PAD (n=7) participants (ANOVA, P=0.082). Approximately 600 fibers were analyzed per subject. Data represented as Mean ± SEM. Scale bar = 50 µm. [file 12967_2016_1030_MOESM4_ESM.tif]

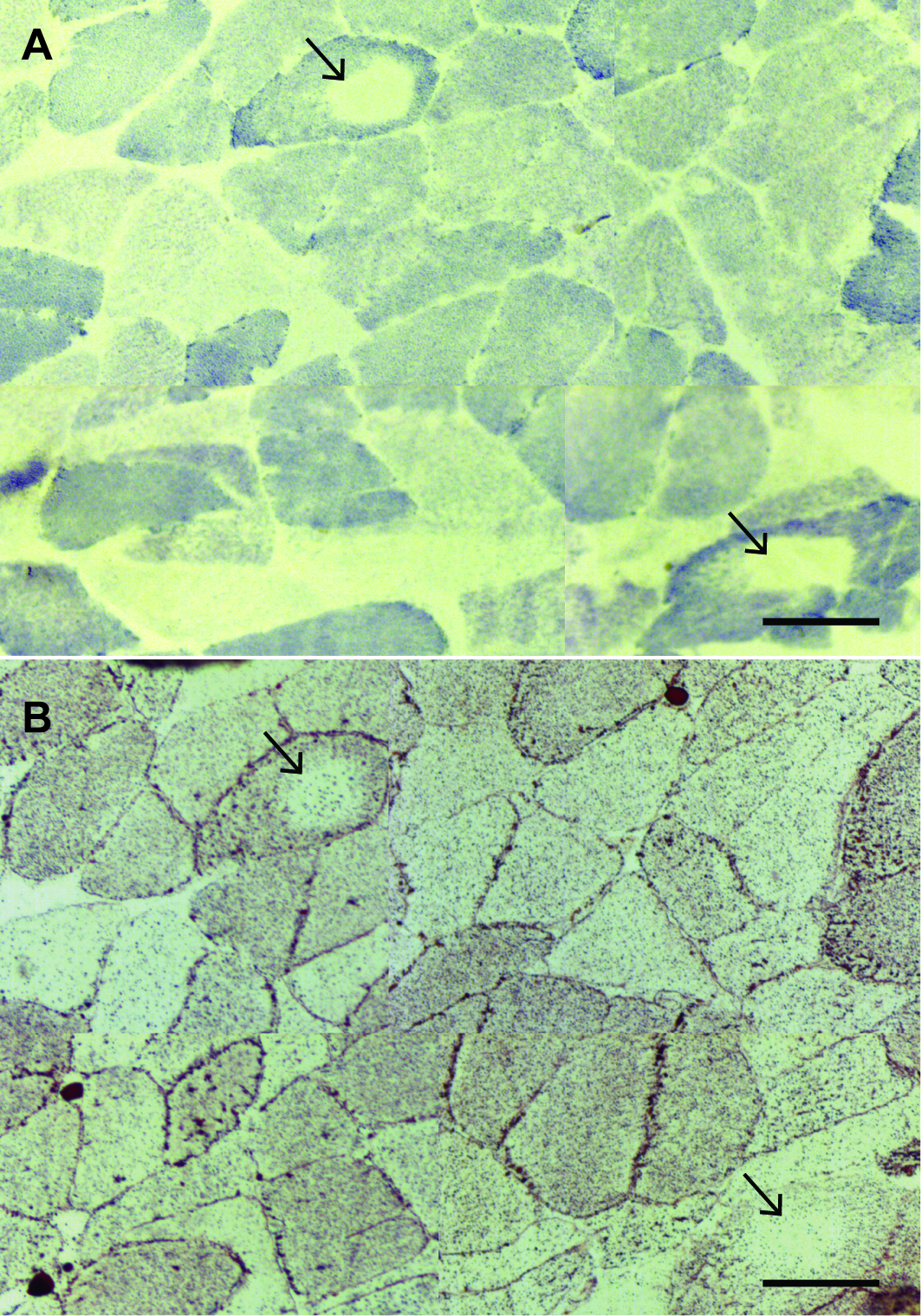

Supplement: Supplementary file 5 — 10.1186/s12967-016-1030-6 Representative images of oil red O (ORO, A) and succinate dehydrogenase activity (SDH, B) staining of gastrocnemius serial sections. Excessive lipid does not accumulate in the SDH cavities. Arrows point to same fibers. Scale bar = 100 µM. [file 12967_2016_1030_MOESM5_ESM.tif]

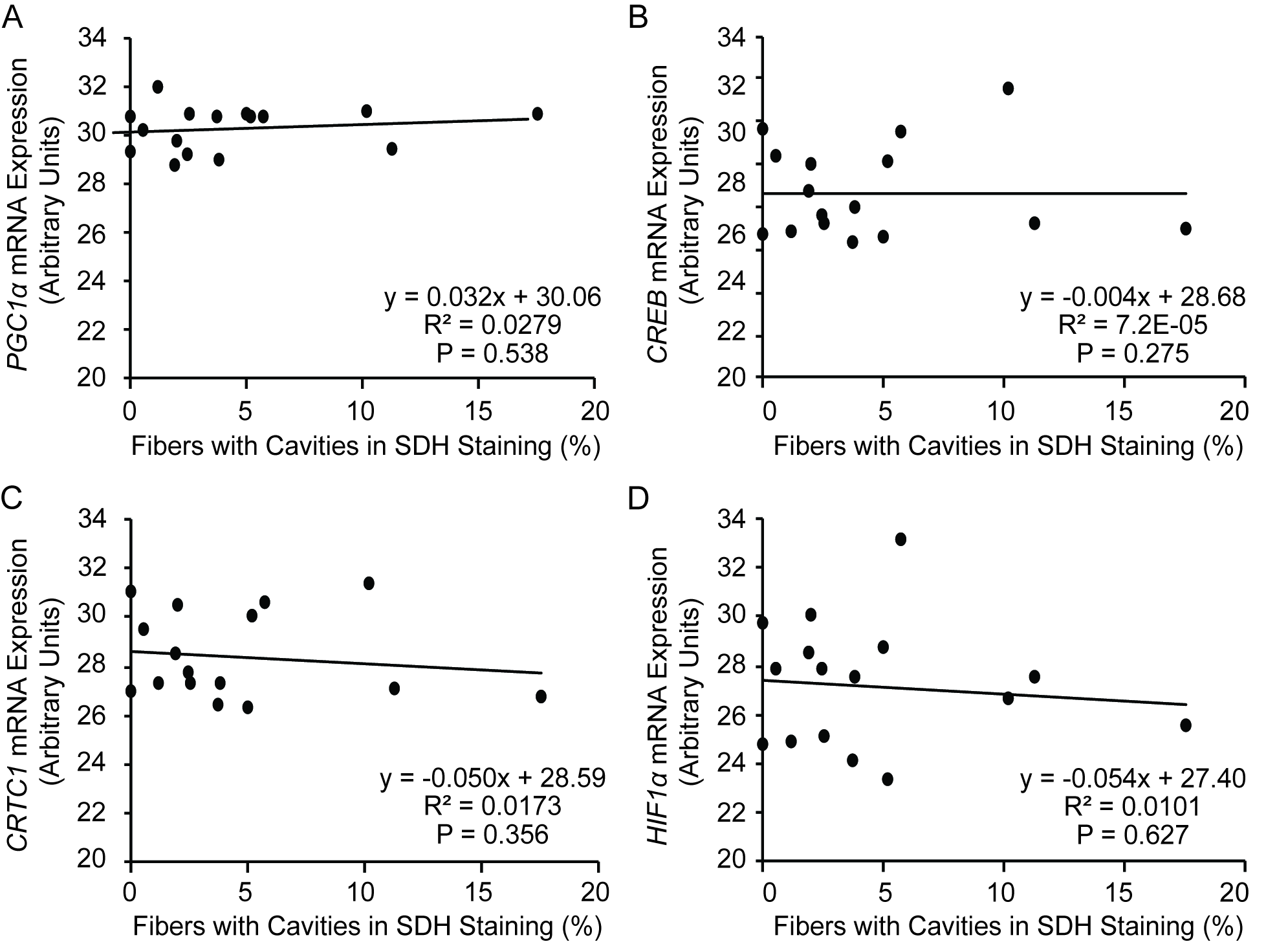

Supplement: Supplementary file 6 — 10.1186/s12967-016-1030-6 Correlations between the percentage of fibers with cavities in SDH staining in PAD gastrocnemius muscle sections (n=18, approximately 1000 fibers analyzed per subject) and PGC1α (A), CREB (B), CRTC1 (C), and HIF1α (D) mRNA expression. [file 12967_2016_1030_MOESM6_ESM.tif]

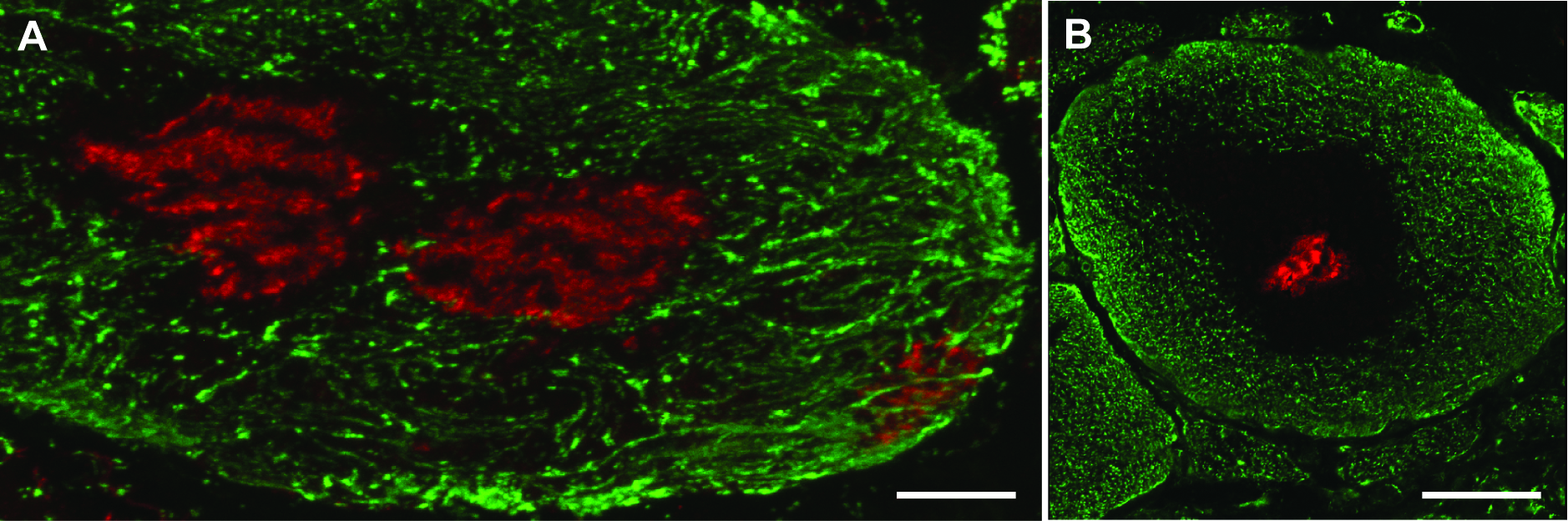

Supplement: Supplementary file 7 — 10.1186/s12967-016-1030-6 Representative confocal images of PAD gastrocnemius muscle sections. A) LC3 (red) is diffuse throughout cavity where mitochondrial cytochrome c oxidase protein (complex IV; COX-1; green) is absent. B) LC3 has formed a plaque in the center of the COX-1 protein cavity. Scale bar = 10 µM. [file 12967_2016_1030_MOESM7_ESM.tif]
